# Non-invasive versus invasive estimation of left ventricular wall stress with cardiac magnetic resonance imaging in severe aortic stenosis

**DOI:** 10.1016/j.jocmr.2025.102016

**Published:** 2025-11-25

**Authors:** Riley J. Batchelor, Stavroula Papapostolou, Jack He, John Kearns, David M. Kaye, Dion Stub, Shane Nanayakkara, Antony Walton, Anoop N. Koshy, Andrew J. Taylor

**Affiliations:** a Department of Cardiology, Alfred Health, Melbourne, Australia; b Department of Cardiology, Royal Melbourne Hospital, Melbourne, Australia; c School of Public Health and Preventive Medicine, Monash University, Melbourne, Australia; d Department of Cardiology, Western Health, Melbourne, Australia; e Department of Aerospace Engineering, Monash University, Melbourne, Australia; f Heart Failure Laboratory, Baker Heart & Diabetes Institute, Melbourne, Australia; g Ambulance Victoria, Melbourne, Australia; h Department of Cardiology, Austin Health, Melbourne, Australia; i Department of Medicine, University of Melbourne, Melbourne, Australia

**Keywords:** CMR, Wall stress, Valvular heart disease

## Abstract

**Background:**

Left ventricular wall stress (LVWS) is a key determinant of myocardial oxygen demand in aortic stenosis (AS), but CMR-based assessment typically requires invasive left ventricular (LV) pressure. We evaluated a fully non-invasive method to quantify systolic LVWS in severe AS.

**Methods:**

In 35 TAVR candidates with severe AS who underwent CMR, circumferential LVWS was calculated using a cylindrical LV model across seven short-axis slices. End-systolic LV pressure was estimated as brachial systolic blood pressure plus echocardiographic mean transvalvular gradient. Circumferential LVWS from this cuff-based approach was compared with LVWS calculated using simultaneously acquired invasive LV pressures. Agreement was assessed using Pearson correlation, linear regression, and Bland–Altman analysis. Six-minute walk test (6MWT) distance was compared between patients above and below the median non-invasive LVWS.

**Results:**

Mean age was 80.1 ± 6.2 years and 74.3% were male. Non-invasive and invasive circumferential LVWS were strongly correlated (r = 0.89, p < 0.001) with a regression slope (β) of 0.94. Bland–Altman analysis showed a small positive bias (+0.65 Pa) and 95% limits of agreement from –9.8 to +11.1 Pa. Patients in the lower 50% of non-invasive LVWS had greater 6MWT distance than those in the upper 50% (median [IQR] 352 [224–383] vs 215 [176–279] m, p = 0.046).

**Conclusions:**

Non-invasive CMR-based estimation of circumferential LVWS using cuff pressure and echocardiographic gradient closely approximates invasively measured LVWS and retains functional discriminative value. This pragmatic approach may facilitate broader research into the application of LVWS as a non-invasive marker of early LV decompensation in AS.

## Graphical abstract

**Figure:**
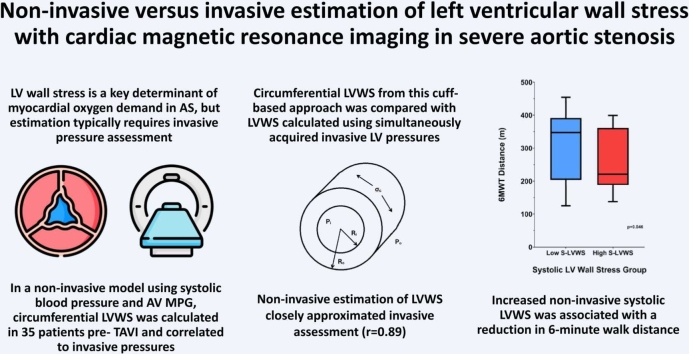

In patients with aortic stenosis (AS), left ventricular wall stress (LVWS) plays a central role in determining myocardial oxygen demand. In a recent study of patients with valvular heart disease (Papapostolou et al. [1]) from our group, we combined high-resolution cardiovasuclar magnetic resonance (CMR) imaging with invasive pressure measurements obtained during valvular intervention to quantify systolic LVWS in a cylindrical model of the left ventricle. In patients with severe AS undergoing transcatheter aortic valve replacement (TAVR), systolic LVWS was significantly elevated at baseline and decreased acutely following TAVR. Notably, higher preprocedural systolic LVWS was independently associated with poorer functional capacity, as reflected by shorter 6-minute walk test distances, and with more advanced structural remodeling—characterized by larger left ventricular (LV) volumes, lower ejection fraction, and greater left atrial dilation [1]. These findings highlighted systolic LVWS as a physiologically relevant marker of early decompensation in pressure-overloaded hearts. Our study relied on invasive LV pressure measurements obtained using 6F pigtail catheters at the time of TAVR, which, while a gold standard in hemodynamic assessment, is resource-intensive, precluding broader application.

Here, we aimed to assess the reliability of quantifying systolic LVWS using the CMR dataset and a non-invasive brachial blood pressure applied to the same CMR-derived cylindrical model, without reliance on invasive catheter data. LV end-systolic pressure was estimated non-invasively by summing the brachial systolic blood pressure and the echocardiographic mean transvalvular gradient [2]. This value was substituted into the cylinder-based formula for circumferential wall stress in place of invasive catheter pressures (Fig. 1) [1], [3].

σ_c_ = ((p_i_ r_i_^2^ − p_o_ r_o_^2^)/(r_o_^2^ − r_i_^2^)) + (r_i_^2^ r_o_^2^ (p_i_ − p_o_)/(r^2^ (r_o_^2^ − r_i_^2^)))·

**Fig. 1:**
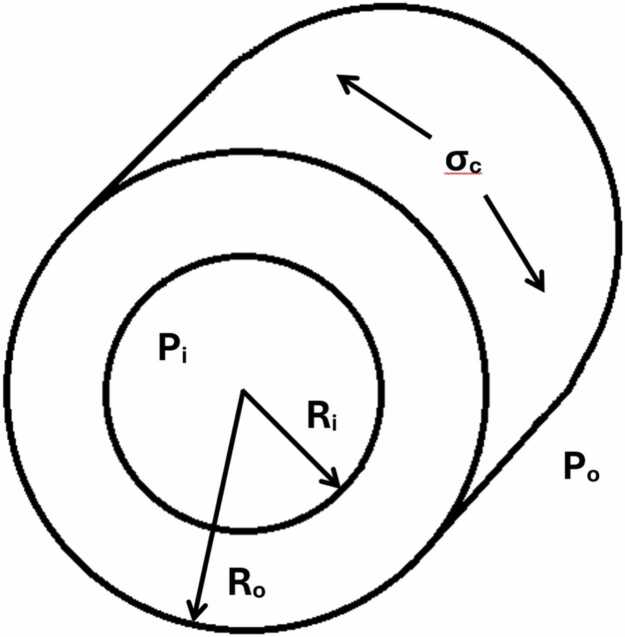
Stress in the circumferential direction (hoop stress) (adapted from The Engineering Toolbox 2005) [3]. *σ_c_* stress in circumferential direction, *p_i_* internal pressure, *p_o_* external pressure (in this model = 0), *r_i_* internal radius, *r_o_* external radius

All patients underwent cine CMR imaging using a 1.5T scanner. LV endocardial and epicardial contours were manually segmented at end-systole across seven short-axis slices, excluding the mitral valve. Eight equally spaced short-axis myocardial segments per slice were analyzed. The internal radius was defined as half the endocardial diameter and the wall thickness as the perpendicular distance between endocardial and epicardial borders at each segment. Circumferential LVWS was computed for each segment using the above equation. Slice-level stresses were averaged to obtain a mean LVWS per slice, and the global systolic LVWS was derived as the mean of all seven slices. As shown in prior work, directional components of LVWS (longitudinal, meridional, and circumferential) were highly correlated; therefore, only circumferential LVWS was recalculated in this analysis [1]. To assess agreement between the invasive and non-invasive methods, both correlation analysis and Bland-Altman analysis were performed. The bias (mean difference) was calculated and plotted alongside 95% limits of agreement. A regression analysis was also performed to determine the slope (β coefficient), which reflects proportional agreement between the two methods. Analyses were conducted using R and RStudio software [4], [5].

From the original TAVR cohort (n = 47), 35 patients had available non-invasive blood pressure data temporally aligned with their echocardiogram. The cohort undergoing non-invasive CMR analysis (n = 35) was representative of an elderly population with severe AS undergoing preprocedural assessment. The mean age was 80.1 ± 6.2 years, and the majority were male (26/35, 74.3%). Cardiometabolic comorbidities were common, with 88.6% (31/35) having hypertension, 47.2% (17/35) documented coronary artery disease (stenosis >50%), and 31.4% (11/35) diabetes mellitus (Table 1). Transthoracic echocardiography, performed within a median 0 days (interquartile range 0–7) of the CMR, confirmed severe AS with a median mean pressure gradient of 42 mmHg and a median indexed aortic valve area of 0.4 cm/m². CMR showed preserved left ventricular ejection fraction (58 ± 12%), elevated indexed LV mass (86.1 ± 16.5 g/m²), and a median indexed left atrial volume of 43 mL/m² (Table 1). A strong linear correlation was noted between invasively and non-invasively derived circumferential LVWS values (Pearson r = 0.89, *p* < 0.001) with a regression slope (β) of 0.94 suggesting proportional agreement (Fig. 2A). A Bland-Altman analysis demonstrated a mean bias of +0.65 Pa, indicating the non-invasive approach slightly overestimates LVWS on average compared with the invasive method. The 95% limits of agreement ranged from −9.8 to +11.1 Pa. These findings support the relative accuracy and acceptable agreement of the non-invasive approach, particularly in the mid-range of LVWS values. Furthermore, when stratified by LVWS values derived from the non-invasive method, patients in the lower 50% of LVWS had significantly greater functional capacity as assessed by the 6-minute walk test compared with those in the upper 50% (median [interquartile range]: 352 [224–383] m vs 215 [176–279] m, *p* = 0.046) (Fig. 3).

**Table 1:** Baseline, TTE, and CMR characteristics.

*Baseline data (n = 35)*	
Age, y	80.1±6.2
Male	26 (74.3%)
BMI, kg/m^2^	28.3±4.5
IHD (coronary stenosis >50%)	17 (47.2%)
Hypertension	31 (88.6%)
Diabetes	11 (31.4%)
Creatinine, µmol/L	84 (71–107)
NYHA functional class I or II	19 (54.3%)
NYHA functional class III or IV	16 (45.7%)
Preprocedural 6MWT, m	280 (202–376)
*TTE data*	
AV MPG, mmHg	42.0 (38.0–52.0)
AV DI	0.21 (0.18–0.24)
AVAi, cm/m^2^	0.4 (0.3–0.4)
*CMR data*	
LV mass indexed, g/m^2^	86.1±16.5
LV EDV, mL	152 (133–175)
LVEF, %Values are expressed as n (%), mean±SD or median (IQR)	58±12
LA biplane indexed, mL/m^2^	43 (35.9–63.1)

*TTE* transthoracic echocardiography, *CMR* cardiovascular magnetic resonance, *BMI* body mass index, *IHD* ischemic heart disease, *NYHA* New York Heart Association, *6MWT* six minute walk test, *LVEF* left ventricular, *EDV* end-diastolic volume, *LVEF* left ventricular ejection fraction, *IQR* interquartile range, *SD* standard deviation, *AV MPG* Aortic valve mean pressure gradient, *AV DI* aortic valve dimensionless index, *AVAi* indexed aortic valve area, *LA* left atrial

Values are expressed as n (%), mean ± SD or median (IQR)

**Fig. 2:**
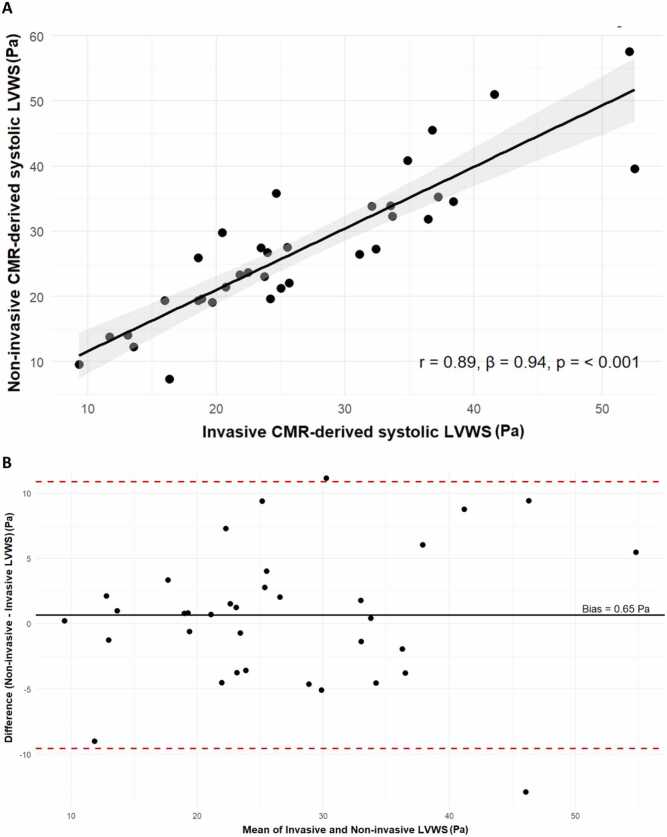
(A) Correlation between systolic LVWS calculated using invasive and non-invasive measurements. (B) Bland-Altman plot comparing the difference in invasive and non-invasive systolic LVWS. *LVWS* left ventricular wall stress, *CMR* cardiac magnetic resonance

**Fig. 3:**
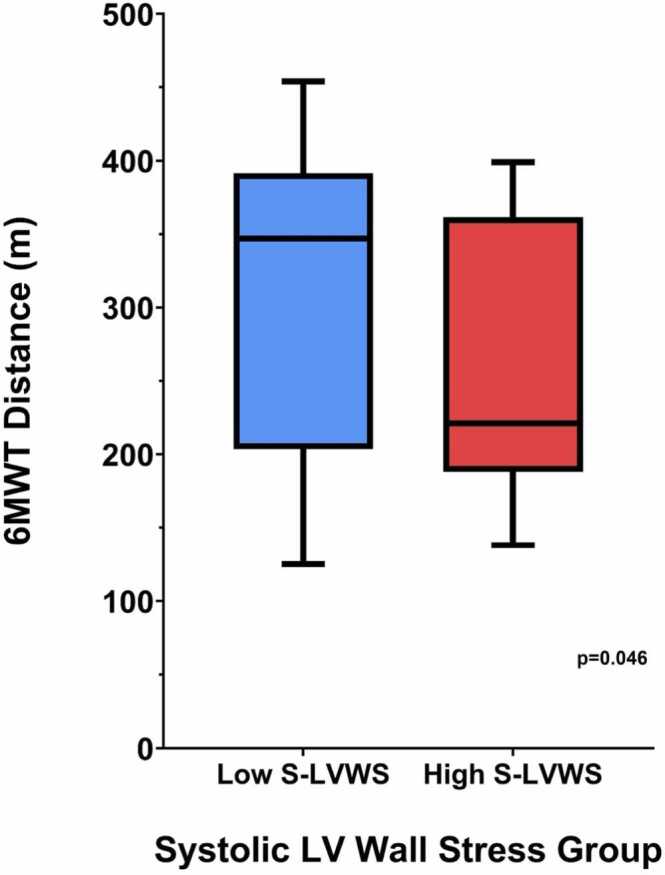
High versus low non-invasive systolic LVWS and preprocedural 6-minute walk test results. *LVWS* left ventricular wall stress, *6MWT* 6-minute walk test, *LV* left ventricular

In the setting of asymptomatic moderate or severe AS, current guidelines rely heavily on echocardiographic indices such as peak velocity, mean gradient, and valve area to guide timing of intervention [6]. However, these parameters are imperfect predictors of disease progression and symptom onset [7]. Recent randomized studies have challenged the paradigm of conservative management in asymptomatic severe AS. In the EARLY TAVR trial, earlier transcatheter valve replacement was associated with improved outcomes, despite the absence of symptoms. Furthermore, approximately one-third of “asymptomatic” patients with severe AS screened had symptoms upon treadmill testing and were therefore excluded [8]. The EVOLVED study recently found that in asymptomatic patients with severe AS and myocardial fibrosis on CMR, aortic valve intervention had no effect on all-cause death or AS-related hospitalization [9]. Our original findings demonstrated that LVWS is independently associated with functional status and remodeling, and that even among patients with similar echocardiographic gradients, wall stress can vary substantially depending on myocardial adaptation. The current analysis suggests that non-invasive LVWS measurement could be incorporated into risk stratification frameworks in future studies.

This analysis has limitations. The sample size was modest (n = 35), and while representative of the broader TAVR population, results may not generalize to all severities of AS. The proportion of female participants in this cohort was low, consistent with our index study, and may limit generalizability of findings to women. In addition, blood pressure measurement was not simultaneous with Doppler assessment, though efforts were made to ensure temporal proximity. Despite this, the strength of correlation observed suggests that the signal is robust and that modest temporal discrepancies appear not to significantly impair accuracy. We used unadjusted brachial systolic pressure in our model, prioritizing a clinically practical and scalable approach that reflects real-world non-invasive assessments, rather than applying central pressure correction factors.

Non-invasive CMR estimation of circumferential LVWS using echocardiographic mean gradient and cuff blood pressure shows strong correlation with invasively measured values. This methodological simplification enables safer and broader study of wall stress physiology in AS, offering scope for further research into non-invasive markers of early LV decompensation, utilizing this methodology to risk-stratify patients who may benefit from more frequent surveillance or earlier intervention.

## Author contributions

**Shane Nanayakkara:** Writing – review & editing, Visualization, Validation. **Dion Stub:** Writing – review & editing, Visualization, Validation, Supervision. **David M. Kaye:** Writing – review & editing, Visualization, Validation, Supervision. **John Kearns:** Methodology, Investigation, Formal analysis. **Anoop N. Koshy:** Writing – review & editing, Validation, Methodology. **Antony Walton:** Writing – review & editing. **Andrew J. Taylor:** Writing – review & editing, Supervision, Methodology. **Jack He:** Writing – review & editing, Visualization, Validation, Investigation. **Stavroula Papapostolou:** Writing – review & editing, Visualization, Validation, Data curation, Conceptualization. **Riley J. Batchelor:** Writing – original draft, Visualization, Validation, Methodology, Investigation, Formal analysis, Data curation, Conceptualization.

## Declaration of competing interests

The authors declare that they have no known competing financial interests or personal relationships that could have appeared to influence the work reported in this paper.
